# Metabolites Reprogramming and Na^+^/K^+^ Transportation Associated With Putrescine-Regulated White Clover Seed Germination and Seedling Tolerance to Salt Toxicity

**DOI:** 10.3389/fpls.2022.856007

**Published:** 2022-03-22

**Authors:** Bizhen Cheng, Muhammad Jawad Hassan, Guangyan Feng, Junming Zhao, Wei Liu, Yan Peng, Zhou Li

**Affiliations:** College of Grassland Science and Technology, Sichuan Agricultural University, Chengdu, China

**Keywords:** growth, metabolome, differentially expressed gene, amylolysis, ionic equilibrium, metabolic pathway

## Abstract

Soil salinization is a serious challenge to many countries worldwide. Putrescine (Put) is related to the improvement of seed germination under salt stress, but molecular and metabolic mechanisms are still not fully understood. The objectives of this study were to determine the effect of seed soaking with Put on germination characteristics under salt stress induced by 100 mm sodium chloride (NaCl) and to further analyze subsequent stress tolerance associated with amylolysis, oxidative damage, sodium (Na^+^)/ potassium (K^+^) accumulation and transportation, and metabolic homeostasis in white clover (*Trifolium repens* cv. Haifa) seedlings. The results showed that seed soaking with Put significantly alleviated salt-induced decreases in the endogenous Put content, germination rate, germination vigor, germination index, Rl/SL, and fresh/dry weight of seedlings. Put application also significantly promoted starch metabolism through activating α-amylase and β-amylase activities under salt stress. The metabolomic analysis showed that seed soaking with Put significantly increased the accumulation of polyamines (Put and spermidine), amino acids (γ-aminobutyric acid, glutamate, alanine, proline, citrulline, etc.), organic acids (ketopentanic acid, malonic acid, malic acid, ketopentanic acid, cis-sinapinic acid, etc.), lipids and fatty acids (glycerol, stearic acid, linoleic acid, palmitic acid, etc.), sugars (levoglucosan, fucose, and anhydro-D-galactose), alcohols (myo-inositol, allo-inositol, hexadecanol, and threitol), and other metabolites (thymine, xanthine, adenine, guanine, and glycerol 1-phosphate, etc.) associated with enhanced tricarboxylic acid (TCA) cycle and γ-aminobutyric acid (GABA) shunt contributing to better osmotic adjustment, cell membrane stability, energy supply, and metabolic homeostasis when seeds germinated under salt stress. In addition, Put significantly up-regulated the *AsSOS1*, *NHX6*, *SKOR*, *HKT1*, and *HKT8* expression levels which played critical roles in Na^+^ rejection and K^+^ retention resulting in higher K^+^/Na^+^ ratio during seed germination under salt stress. The Put-induced up-regulation of *HAL2* transcription level could reduce the toxicity of 3′-phosphoadenosine-5′-phosphosulfate (PAPS) in cells. Current findings will provide an integrative understanding of Put-induced salt tolerance associated with amylolysis, metabolic regulation, and ionic homeostasis during seed germination.

## Introduction

Salt stress is one of the most drastic abiotic stresses, especially in arid and semi-arid regions globally (Bouaziz et al., 2020). About 17.5% of the total agricultural lands in more than 100 countries are severely affected by salt stress (Food and Agriculture Organization [FAO], 2021). Salt stress disrupts various physiological functions during all phases of the plant life cycle including seed germination, seedling growth and development, and flowering (Parihar et al., 2015). In addition, salt stress is also responsible for physiological drought, as large amounts of metal ions in the rhizosphere seriously reduce soil water potential and inhibit the water absorption capacity of the roots (Yadav et al., 2011). It is well known that seed germination is not only the basic foundation but also the most sensitive and imperative phase of the plant life cycle (Cuartero et al., 2006). Previous studies have found that salt stress significantly reduced the germination of many important forage species including white clover (*Trifolium repens*), alfalfa (*Medicago sativa*), and oats (*Avena sativa*) (Hu et al., 2006; Wu et al., 2009; Cheng et al., 2018). A previous study has reported that salt stress resulted in massive accumulation of sodium (Na^+^) which causes ion toxicity, mineral nutrition disorder, and metabolic imbalance in white cover seedlings (Cheng et al., 2018). During germination under salt stress, seeds often experience imbalance in active oxygen metabolism leading toward excessive production of reactive free radicals responsible for oxidative damage to various cell organelles, retardation in seed germination, and even seed mortality (Cheng et al., 2018; Luo et al., 2020). In addition, the accumulation of Na^+^ in germinating seeds prevents the mobilization of starches, amino acids (AAs), and sugars which are essential for the synthesis of structural tissues as well as proteins in embryos, thus inhibiting seed germination (Prakash et al., 1988; Ramagopal, 1990; Cheng et al., 2018).

When plants are exposed to salt toxicity, the absorption, efflux, and compartmentalization of Na^+^ are vital physiological processes involved in salt tolerance. Salt stress activates calcium ion (Ca^2+^) to regulate the salt overly sensitive (SOS) signaling pathway which serves as one of the vital pathways for Na^+^ excretion in plants (Gong et al., 2020). Vacuolar H^+^-ATPase (H^+^-ATPase) in the plasma membrane hydrolyzes ATP to pump H^+^ from the cytoplasm into the vacuole. This process generates electrochemical gradient and proton driving force, hence driving SOS1-regulated Na^+^ excretion from the cytoplasm into the intercellular space (Blumwald, 2000). The Na^+^ (K^+^)/H^+^ antiporters (*NHX*s) gene family has also been found to be involved in salt stress response, cell expansion, vesicle transport, and pH homeostasis (Bassil et al., 2012; Bassil and Blumwald, 2014). NHX6, which is located in the Golgi and trans Golgi network (TGN), compartmentalizes cytoplasmic Na^+^ into the vacuole, thereby improving osmotic adjustment and also avoiding Na^+^ toxicity in the cytosol (Wang et al., 2015). Moreover, high-affinity potassium transporters (HKTs) are Na^+^ transporters that regulate the long-distance transport of Na^+^ from the roots to the leaves and reduce the toxicity of Na^+^ to aerial parts (Almeida et al., 2017). However, some members of HKTs also possess the characteristics of potassium (K^+^) transporters associated with the maintenance of K^+^ homeostasis in plants under salt stress (Hauser and Horie, 2010). Stelar K^+^ outward rectifier (SKOR) is one of the outward-rectifying shaker K^+^ channels mediating release and transportation of K^+^ from the xylem to the shoot (Huang et al., 2018). Yellow halotolerant protein 2 (HAL2) performs vital roles in sulfur assimilation and RNA processing, and *HAL2* overexpression can increase tolerance of tomato (*Lycopersicon esculentum*) to high Na^+^ concentration. Under salt stress, the decline in HAL2 activity significantly inhibits the sulfur assimilation pathway, resulting in delayed plant growth (Arrillaga et al., 1998). A previous study has demonstrated that improved endogenous polyamines (PAs) including putrescine (Put), spermidine (Spd), and spermine (Spm) levels induced by exogenous chitosan significantly alleviated salt damage associated with the significant up-regulation of *NHX4*, *NHX5*, *NHX6*, and *SOS1* gene expression in leaf and root of creeping bentgrass (*Agrostis stolonifera*) (Geng et al., 2020).

Polyamines (PAs) are important members of aliphatic nitrogenous bases existing widely in different plant and animal species. In plants, Put, Spd, and Spm are three main types of PAs (Kusano et al., 2008). PAs exhibit multiple beneficial physiological effects on alleviating salt damage by mediating osmotic potential, ion balance, and antioxidant defense system (Kasukabe et al., 2004; Verma and Mishra, 2005; Sudhakar et al., 2015). Kasukabe et al. (2004) found increased endogenous Spd content in transgenic *Arabidopsis* overexpressing an *FSPD1* enhanced salt tolerance. Many studies have shown that exogenous Spd and Spm application improved the activities of antioxidant enzymes including ascorbate peroxidase (APX) and catalase (CAT), thereby decreasing oxidative damage of organisms under saline conditions (Roychoudhury et al., 2011; Sung et al., 2011). In addition, Put, Spd, and Spm could significantly mitigate salt-induced declines in seed germination rate, root or stem elongation, and fresh weight associated with the improvement of antioxidant capacity and reduced reactive oxygen species (ROS) accumulation during seed germination (Prakash et al., 1988; Benavides et al., 1997; Çavuşoğlu et al., 2007). The study of Çavuşoğlu et al. (2007) also found that Put pretreatment significantly improved the seed germination rate, fresh or dry weight, and root length or stem length of barley (*Hordeum vulgare*) seedlings under salt stress. The catabolism of seed storage substances such as starch could be significantly improved by the Put pretreatment contributing to better seed germination and seedling growth of rice (*Oryza sativa*) in response to salt stress (Prakash et al., 1988). Furthermore, increased endogenous Put content by exogenous Put application effectively alleviated adverse effects of salt stress on seed germination and early growth of belladonna (*Atropa belladonna*) seedlings associated with the reduced accumulation of net Na^+^ and chlorine (Cl^–^) in different organs (Benavides et al., 1997). These previous studies indicated the positive roles of Put in plant response to salt stress. However, the regulatory roles of the Put in metabolic homeostasis, Na^+^ and K^+^ transportation, and other potential mechanisms demand further investigation during seed germination under high salt conditions.

White clover, an important legume forage, is cultivated globally due to its low crude fiber as well as high crude protein and nutrient contents. In addition, white clover has also been widely used as an essential ornamental legume or ground-cover plant, hence contributing to the aesthetic value of landscapes. However, salt stress limits its yield and quality. The objectives of this study were (1) to examine seed germination regulated by Put in relation to cell membrane stability, starch metabolism, and Na^+^/K^+^ transport under salt stress; (2) to further explore metabolic homeostasis and metabolites reprogramming associated with alteration in comprehensive metabolites during seed germination based on the analysis of metabonomics in response to salt stress. Present findings will provide new insights about the effects and regulatory mechanisms of Put in legume species during seed germination under salt stress.

## Materials and Methods

### Plant Materials and Treatments

Seeds of white clover (*Trifolium repens* cv. Haifa) were surface-sterilized with 0.1% HgCl_2_ for 5 min and rinsed four times with distilled water. For the soaking pretreatment, the seeds were divided into two groups. One group of seeds was soaked in distilled water as the control while the other group of seeds was soaked in Put (30 μM) for 2 h at 20°C, respectively. The soaked seeds were then germinated in Petri dishes containing three sheets of filter papers moistened initially with 10 ml of distilled water or 100 mg⋅l^–1^ sodium chloride (NaCl) and each treatment was replicated four times (50 seeds for each duplicate). The Petri dishes were kept in a growth chamber programmed at average day/night temperature of 23/19°C, 75% relative humidity, and 600 μmol⋅m^–2^⋅s^–1^ photosynthetic photon flux density for 7 days. Filter papers, NaCl solution, and distilled water were refreshed every day. The seeds were sampled after 7 days of germination for various biochemical, physiological, and metabolomics analyses.

### Determination of Seed Germination Characteristics

Germination vigor (GV) and germination percentage (GP) were evaluated after 3 or 7 days of germination, respectively. The germination index (GI) and mean germination time (MGT) were calculated according to the following formulas:


(1) $$G\,I=\sum \frac{G\,t}{T\,t}$$

where Gt is the number of the germinated seeds in the t days; Tt is the time corresponding to Gt:


(2) $$M\,G\,T=\frac{\sum T\,i\times N\,i}{\sum N\,i}$$

where Ni is the number of the newly germination seeds in times of Ti, respectively (Zhang et al., 2007).

The root length (RL), shoot length (SL), seedling fresh weight (FW), seedling dry weight (DW), and seed vigor index (VI) were measured after 7 days of germination. The VI was calculated based on the formula VI = FW × GI (Li et al., 2014).

### Determination of Starch Content and Amylase Activities

For starch content, dry powders (0.05 g) were mixed with 3 ml of ethanol (80%) and the mixture was heated in the water bath at 80°C for 30 min. After being centrifuged at 12,000 *g* for 10 min, the supernatant was removed and 2 ml of distilled water was added. Later, the centrifuge tubes were boiled in a water bath at 80°C for 15 min, and then 4 ml of 9.2 mM HClO_4_ was added and gently shaken for 15 min. After centrifugation at 12,000 *g* for 15 min, 1 ml of supernatant was dissolved in 2 ml of anthrone sulfuric acid, and the mixture was boiled in a water bath for 10 min. The absorbance of the solution was measured at 620 nm (Smith, 1969). Amylase activity was determined following the methods of Kishorekumar et al. (2007) with some modifications. Fresh seedlings (0.1 g) were homogenated in distilled water (1.5 ml) and centrifuged at 12,000 *g* for 25 min. A total of 0.5 ml of supernatant was incubated at 70°C for 15 min, and then 0.5 ml of citrate buffer (0.1 mm) and 1 ml of 1% soluble starch solution were added. After being incubated at 30°C for 5 min, the mixture was heated at 40°C for 15 min. The α-Amylase activity was estimated spectrophotometrically at 540 nm. For β-amylase activity, the supernatant was inactivated initially at pH 3.4, later β-amylase activity was detected using the same method mentioned above for α-amylase activity.

### Determination of Oxidative Damage and Membrane Stability

To analyze the malonaldehyde (MDA) content, fresh seedlings (0.2 g) were homogenated with 50 mm cold phosphate buffer (1 mL, pH 7.8). After this, the homogenate was centrifuged at 12,000 g at 4°C for 30 min to get the supernatant. A 0.5 ml of supernatant was mixed with 1 ml of the reaction solution [20% W/V tricarboxylic acid (TCA) and 0.5% W/V thiobarbituric acid (TBA)] and then the mixture was placed in a water bath at 95°C for 30 min. After being centrifuged at 12,000 g for 10 min, the absorbance of the supernatant was detected at 532 and 600 nm (Dhindsa et al., 1981). The hydrogen peroxide (H_2_O_2_) content was assayed by using the potassium iodide (KI) method. The oxidation product was measured at 390 nm (Velikova et al., 2000). For the relative electrical conductivity (EC), the fresh seedlings (0.1 g) were immersed in 15 ml of deionized water for 12 h, and then the initial conductivity of the solution (C_initial_) was measured by using a conductivity meter (DDS-307A, Shanghai Precision Scientific Instrument Co., Ltd., Shanghai, China). The seedlings were killed by autoclaving at 140°C for 30 min and the maximum conductivity of the solution (C_max_) was measured. The percentage of C_initial_ and C_max_ (EC = (C_initial_/C_max_) × 100%) was calculated as the relative EL of seedlings (Blum and Ebercon, 1981).

### Na^+^/K^+^ Content and Genes Expression Analysis

Dry powdered samples (0.3 g) and 5 ml of nitric acid were added into a Teflon digestion inner tank overnight and placed in a constant temperature drying oven at 80°C for 1.5 h, 120°C for 1.5 h, and then at 160°C for 4 h, respectively. After all acids in the tank evaporated, the residues were washed with 1% nitric acid solution thrice to get a constant volume (25 ml) for the estimation of Na and K contents (Geng et al., 2021). The Na and K contents in the solution were then determined by using the American thermoelectric inductively coupled plasma (ICP) inductively coupled plasma emission spectrometer (ICAP6300). The transcript levels of genes (Table 1) were performed using a real-time quantitative PCR (qRT-PCR). The assay methods in detail have been described in our previous study (Livak and Schmittgen, 2001). *Trβ-action* was used as the reference gene (Cheng et al., 2018).

**TABLE 1:** Primer sequences and their corresponding GeneBank accession numbers of analyzed genes.

Target gene	Accession no.	Forward primer (5′–3′)	Reverse primer (5′–3′)	Tm/°C
*VP1*	MF405364	GTCCAATCAGTGACAATGCCG	AGAGGGCAAGAGACACAAGAGC	58
*HKT1*	MF405365	TGCATCACCGAAAGACAAAGC	ATCGACAACCCTACATTCCCATA	57
*HKT8*	MF405366	TTCAAGACACGCTGGAGAAACTAT	CGATGGCAGGAATGAGGTGT	57
*SKOR*	MF405367	GTTTCATTTGATATGGTTCTCGGTG	GGCCCTTTATTTGTTCACGGA	58
*HAL2*	MF405368	TTGTGAACCAGTTGAGAAGGCC	TCGGCATCTCCACGACCTATT	61
*H^+^-ATPase*	MF405369	CGTATAGTGTTTGGCTTCATGTTCA	AATGGAGATGGCACCACCCTA	60
*SOS1*	MF405370	TGGTCCATCTGAAAGTGACAATAAC	TCATCAAGCATCTCCCAGTAAGC	57
*NHX6*	MF405371	CAGTCTGGTTTCAGTCTTGCTCC	ACCAAACATCAGGCACTCAACA	60

### Analysis of Metabolomics

The concentration of various metabolites was detected by using a gas chromatography-time of flight mass spectrography (GC-TOFMS). The method of Li et al. (2019) was used for metabolite extraction, separation, and quantification as described clearly in our previous study.

### Statistical Analysis

The data were analyzed by using SPSS 20 (IBM, Armonk, NY, United States). Significant relationships among the treatments were detected by using the LSD at *P* ≤ 0.05.

## Results

### Effect of Put on Seed Germination Characteristics

Salt stress caused significant declines in GP, GV, GI, and VI, whereas MGT increased when compared with the control. Under normal conditions, Put treatment did not exhibit significant effects on GV, GP, GI, and MGT. Under salt stress, seeds primed with Put exhibited significantly higher GV, GP, and GI than untreated seeds. The GV, GP, GI, or VI of seeds soaked in Put were increased by 17, 13.5, 37.8, or 134.5% than that of seeds soaked in water under salt stress, respectively (Table 2).

**TABLE 2:** Effects of seed priming with water or putrescine (Put) on seed germination characteristics.

Treatment	GP (%)	GV (%)	GI	MGT (d)	VI
Control	96.00 ± 2.83a	95.00 ± 1.15a	36.17 ± 0.95a	1.53 ± 0.08b	1.48 ± 0.12a
Put	98.00 ± 1.63a	95.50 ± 1.91a	36.04 ± 1.32a	1.63 ± 0.16b	1.48 ± 0.10a
NaCl	49.00 ± 2.58c	33.00 ± 1.15c	10.26 ± 0.56c	3.11 ± 0.17a	0.25 ± 0.03c
NaCl+Put	66.00 ± 2.83b	46.50 ± 1.91b	14.13 ± 0.83b	2. 90 ± 0.21a	0. 59 ± 0.03b

*Values are means ± SE (n = 4). Different letters in vertical columns indicate significant differences among various treatments (Control, Put, NaCl, and NaCl+Put). LSD (P ≤ 0.05).*

**FIGURE 1:**
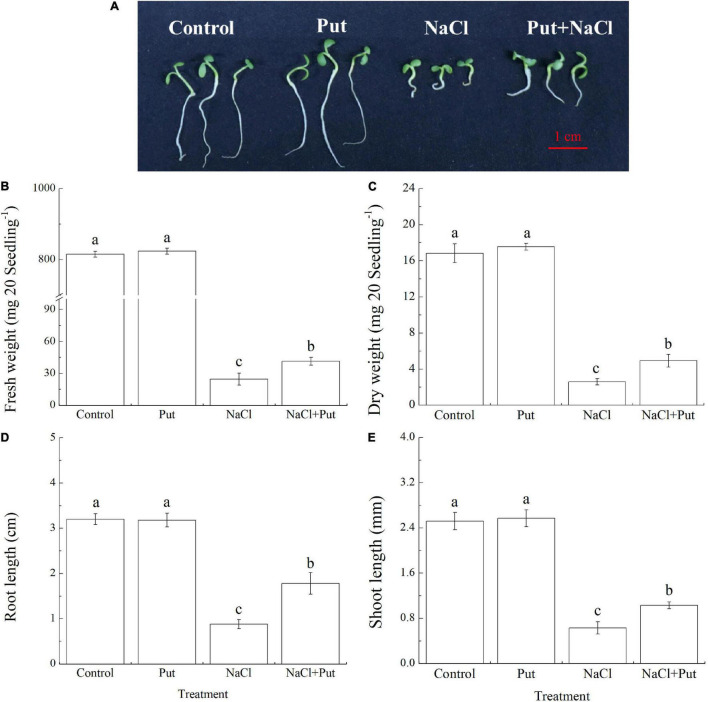
Effects of seed soaking with Put or water on **(A)** phenotypic changes, **(B)** fresh weight, **(C)** dry weight, **(D)** root length, and **(E)** shoot length of white clover seedlings after 7 days of germination under normal condition and salt stress. Vertical bars indicate ± SE of means (*n* = 4). Different letters above columns indicate significant differences. LSD (*P* ≤ 0.05).

Figure 1A shows the phenotypic differences between Put-pretreated and non-pretreated seeds after 7 days of germination. The FW, DW, RL, and SL of the seedlings did not show significant differences between the “Control” and the “Put” treatment under normal conditions (Figures 1B,C). Salt stress significantly decreased the FW, DW, RL, and SL of untreated seeds, however, the seeds primed with Put exhibited significantly higher FW, DW, RL, and SL after 7 days of germination under salt stress, as reflected by a 68.56, 90.73, 102.27 or 63.49% increase in FW, DW, RL or SL, respectively (Figures 1B–E).

### Effect of Put on Oxidative Damage, Membrane Stability, and Starch Metabolism

**FIGURE 2:**
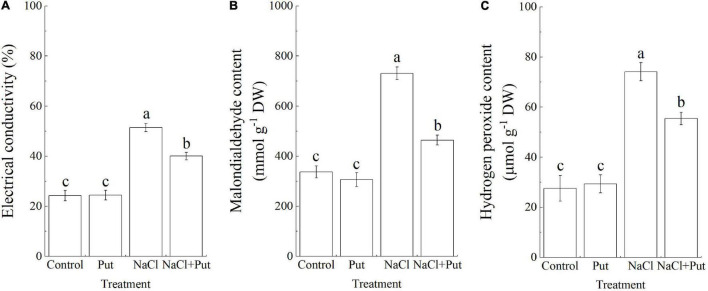
Effects of seed soaking with Put or water on **(A)** electrical conductivity, **(B)** malondialdehyde content, and **(C)** hydrogen peroxide content of white clover seedlings after 7 days of germination under normal condition and salt stress. Vertical bars above columns indicate the standard error of each mean (*n* = 4). Different letters indicate a significant difference (*P* ≤ 0.05).

Salt stress leads to the overaccumulation of ROS which causes oxidative damage to cells. The EC and MDA content are important indicators of cell membrane stability and oxidative damage in plants (Liu et al., 2007; Cheng et al., 2018). Salt stress caused significant increases in EC, MDA, and H_2_O_2_ content (Figure 2). The EC, MDA, and H_2_O_2_ content of Put-treated or untreated seedlings did not show any significant difference under normal conditions, but seeds soaked with Put exhibited significantly lower EC, MDA, and H_2_O_2_ content when compared with water primed seeds under salt stress (Figures 2A–C).

**FIGURE 3:**
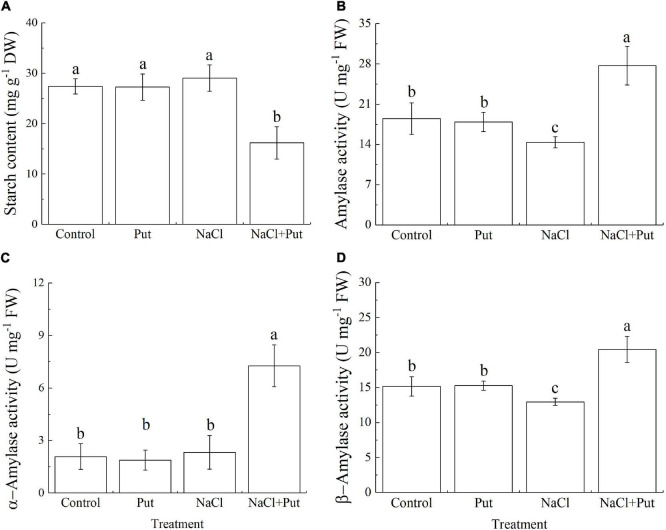
Effects of seed soaking with Put or water on **(A)** starch content, **(B)** amylase activity, **(C)** α-amylase activity, or **(D)** β-amylase activity of white clover seedlings after 7 days of germination under normal condition and salt stress. Vertical bars above columns indicate the standard error of each mean (*n* = 4). Different letters indicate a significant difference (*P* ≤ 0.05).

Starch is the main energy reserve closely related to seed germination and stress resistance (Yang et al., 2016). Salt stress significantly decreased the germination and embryo growth of white clover seeds associated with significant inhibition of starch catabolism through decreasing amylase activity such as α-amylase activity or β-amylase activity (Cheng et al., 2018). The Put application did not have significant effects on starch content, amylase activity, α-amylase activity, and β-amylase activity during seed germination under normal conditions (Figures 3A–D). In addition, salt stress showed no impact on starch content and α-amylase activity in seedlings without Put pretreatment, but significantly reduced the starch content and improved amylase activity, α-amylase activity, or β-amylase activity in seedlings pretreated with the Put (Figures 3A–D).

### Effect of Put on Na^+^/K^+^ Content and Genes Involved in Na^+^/K^+^ Transportation

**FIGURE 4:**
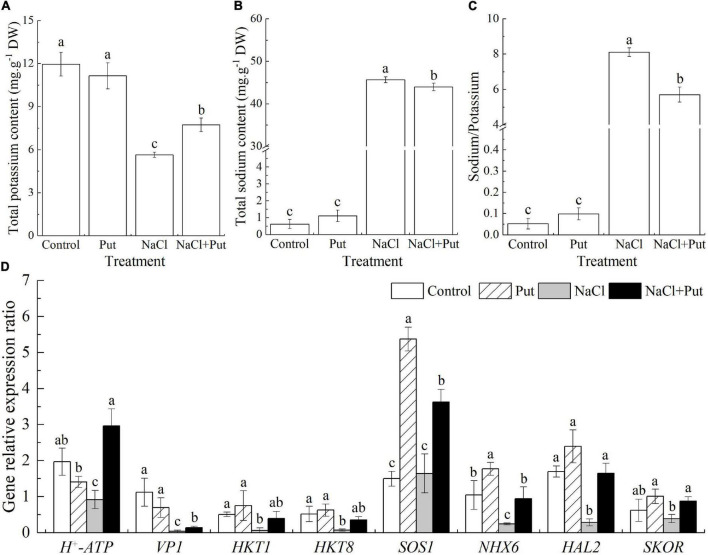
Effects of seed soaking with Put or water on **(A)** total sodium content, **(B)** total potassium content, **(C)** sodium/potassium ratio, and **(D)** relative expression of genes involved in sodium and potassium transportation (*VP1*, *HKT1*, *HKT8*, *SKOR*, *HAL2*, *H^+^-ATPase*, *SOS1*, and *NHX6*) of white clover seedlings after 7 days of germination under normal condition and salt stress. Vertical bars above columns indicate the standard error of each mean (*n* = 4). Different letters indicate a significant difference (*P* ≤ 0.05). The relative expression of genes is based on the *Trβ-action* gene expression.

The Put treatment did not affect the Na^+^ and K^+^ content and Na^+^/K^+^ ratio when the seeds germinated under normal conditions (Figures 4A–C). Salt stress significantly reduced the K^+^ content and improved the Na^+^ content as well as the Na^+^/K^+^ ratio (Figures 4A–C). However, seeds soaked with Put maintained significantly higher K^+^ content and lower Na^+^ content or Na^+^/K^+^ ratio than untreated seeds after 7 days of germination under salt stress (Figures 4A–C). Under normal conditions, the transcript levels of *H^+^-ATPase*, *Vacuolar H^+^ pyrophosphatase 1 (VP1)*, *HKT1*, *HKT8*, *HAL2*, and *SKOR* were not significantly affected by the Put pretreatment, but the Put significantly up-regulated the *SOS1* and *NHX6* expression (Figure 4D). Salt stress had no significant effects on *SOS1* and *SKOR* expression but decreased the transcript levels of all other genes (*HKT1*, *HKT8*, *HAL2*, *H^+^-ATPase*, *VP1*, *NHX6*) under test (Figure 4D). The Put-pretreated seedlings exhibited 3.23, 3.65, 6.20, 4.94, 2.20, 3.90, 5.77, or 2.25 times higher transcript levels of *H^+^-ATPase*, *VP1*, *HKT1*, *HKT8*, *SOS1*, *NHX6*, *HAL2*, or *SKOR* when compared with water primed seeds under salt stress, respectively (Figure 4D).

### Effect of Put on Metabolic Profiles

**FIGURE 5:**
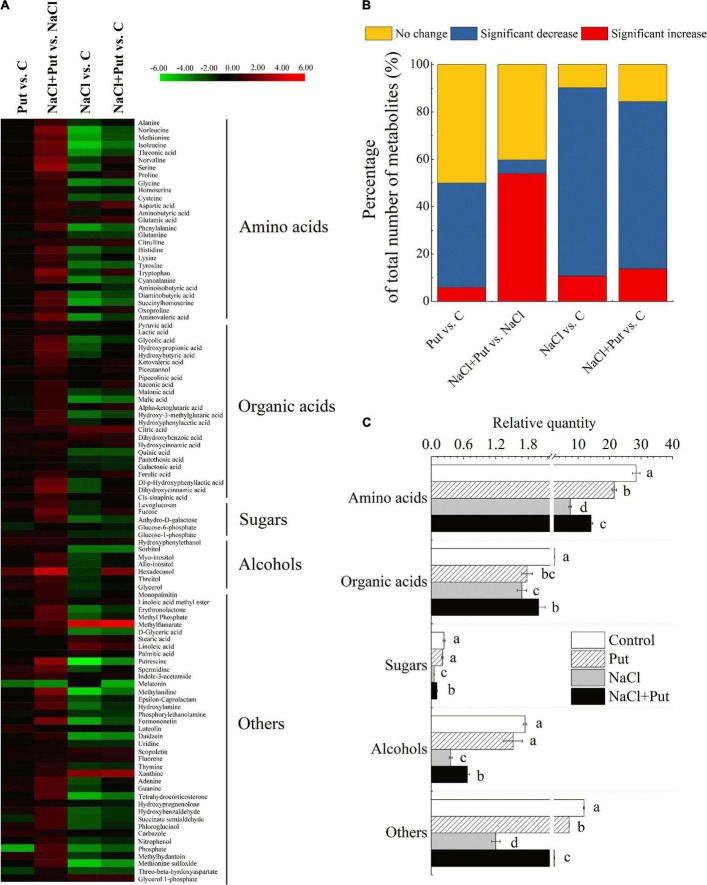
Effects of seed soaking with Put or water on **(A)** heat map of changes in 102 metabolites, **(B)** percentage of the total number of metabolites in each group, and **(C)** total content of AAs, organic acids, sugars, alcohols, and others in each treatment in white clover seedlings after 7 d of germination in response to exogenous Put application and salt stress. Log_2_ (fold change) ratios were shown in the heat map. Red and green indicate the up—and down—regulation, respectively. Vertical bars above columns indicate the standard error of each mean (*n* = 4). Different letters indicate a significant difference. LSD (*P* ≤ 0.05).

More than 1,000 peaks were detected by GC-TOFMS and over 107 putative metabolites were found in seedlings of white clover. A total of 102 differentially expressed metabolites (DEMs) were identified and quantified in four different comparison groups (Put vs. C, Put+NaCl vs. NaCl, NaCl vs. C, and NaCl + Put vs. C) including 27 AAs, 24 organic acids, 10 lipids, and fatty acids, 5 sugars, 6 alcohols and 30 other metabolites (Figure 5A). The heat map showed an overall change of 102 DEMs commonly or differentially regulated by Put and NaCl (Figure 5A). A 44.12, 5.88, 79.41, or 70.59% metabolites significantly decreased in the Put vs. C, NaCl+Put vs. NaCl, NaCl vs. C, or NaCl+Put vs. C, respectively (Figure 5B). A total of 53.92% metabolites significantly increased in the NaCl+Put vs. NaCl, and only 5.88, 10.78, or 13.73% metabolites significantly increased in Put vs. C, NaCl vs. C, or NaCl+Put vs. C, respectively (Figure 5B). The total content of AAs, organic acids, sugars, alcohols, and others decreased significantly in both water-primed and Put-primed seeds under salt stress, as shown in Figure 5C. However, seeds soaked with Put exhibited an 88.89, 18.49, 91.85, 86.54, or 98.18% increase in AAs, organic acids, sugars, alcohols, or others than the seeds soaking with water under salt stress, respectively. The Put pretreatment exhibited no significant effects on the total content of sugars and alcohols, but a reduced accumulation of AAs, organic acids, and others under normal conditions (Figure 5C).

**FIGURE 6:**
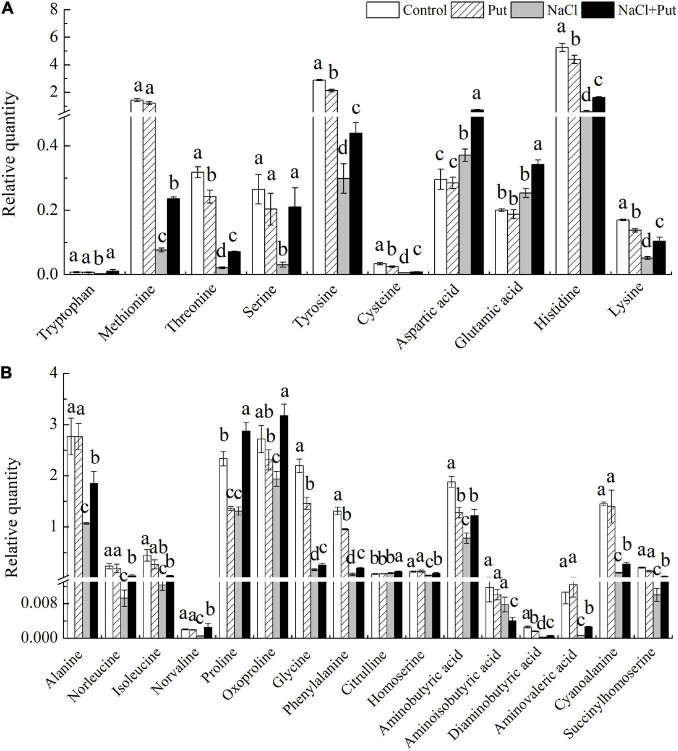
Effects of seed soaking with Put or water on **(A)** relative polar and **(B)** non-polar AAs content in white clover seedlings after 7 d of germination under normal condition and salt stress. Vertical bars above columns indicate the standard error of each mean (*n* = 4). Different letters indicate a significant difference (*P* ≤ 0.05).

The tryptophan, methionine, serine, aspartic acid, glutamic acid, alanine, norleucine, isoleucine, norvaline, oxoproline, citrulline, homoserine, aminoisobutyric acid, aminovaleric acid, cyanoalanine, and succinylhomoserine content were not significantly affected by the exogenous Put under normal conditions, whereas the Put pretreatment significantly decreased the threonine, tyrosine, cysteine, histidine, lysine, proline, glycine, phenylalanine, aminobutyric acid, or diaminobutyric acid content in seeds after 7 days of germination under normal condition (Figures 6A,B). Salt stress had no impact on glutamic acid, citrulline, and aminoisobutyric acid content, but significantly improved aspartic acid content (Figures 6A,B). Except the aminoisobutyric acid, 10 polar AAs (tryptophan, methionine, threonic acid, serine, tyrosine, cysteine, aspartic acid, glutamic acid, histidine, and lysine) and 16 non-polar AAs (alanine, norleucine, isoleucine, norvaline, proline, oxoproline, glycine, phenylalanine, citrulline, homoserine, aminobutyric acid, aminoisobutyric acid, diaminobutyric acid, aminovaleric acid, cyanoalanine, succinylhomoserine) contents were significantly increased in the “NaCl+Put” when compared with “NaCl” (Figures 6A,B).

**FIGURE 7:**
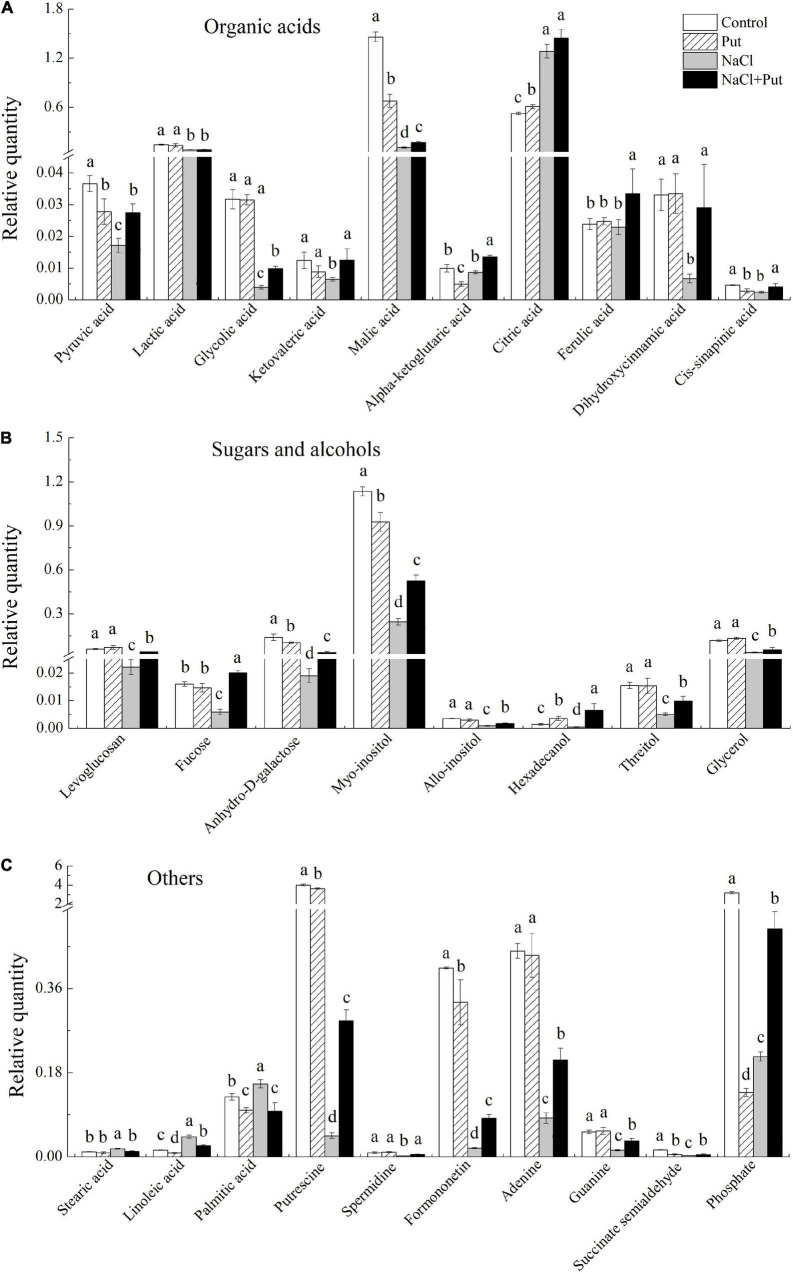
Effects of seed soaking with Put or water on **(A)** organic acids, **(B)** sugars and alcohols, and **(C)** other metabolites content of white clover seedlings after 7 d of germination under normal condition or salt stress. Vertical bars above columns indicate the standard error of each mean (*n* = 4). Different letters indicate a significant difference (*P* ≤ 0.05).

The Put treatment significantly reduced the pyruvic acid, malic acid, α-ketoglutaric acid, and cis-sinapinic acid contents with significant improvement in citric acid contents, whereas other organic acids remained unaffected under normal conditions (Figure 7A). Salt stress had no impact on α-ketoglutaric acid, dihydroxycinnamic acid, and ferulic acid contents with a considerable increase in the citric acid content and a significant reduction in other 7 organic acids contents. However, the seeds soaked with Put maintained a significantly higher 9 organic acids content than the seeds primed with water under salt stress (Figure 7A). Under normal conditions, the Put treatment significantly reduced the anhydro-D-galactose and myo-inositol content with marked improvement in hexadecanol content, while other metabolites remained unaffected (Figure 7B). Salt stress significantly reduced the levoglucosan, fucose, anhydro-D-galactose, myo-inositol, allo-inositol, hexadecanol, threitol, and glycerol contents (Figure 7B). Significantly higher levoglucosan, fucose, anhydro-D-galactose, myo-inositol, allo-inositol, hexadecanol, threitol, and glycerol contents were observed in the seeds primed with Put in contrast to water primed seeds under salt stress (Figure 7B). Under normal conditions, the Put treatment significantly reduced the linoleic acid, palmitic acid, Put, formononetin, succinate semialdehyde, and phosphate content, but had no significant effects on other metabolites (Figure 7C). As compared to the seeds soaked with water, the seeds primed with Put exhibited significantly higher Put, Spd, formononetin, adenine, guanine, succinate semialdehyde, and phosphate content, but had significantly lower stearic acid, linoleic acid, and palmitic acid content under salt stress (Figure 7C).

**FIGURE 8:**
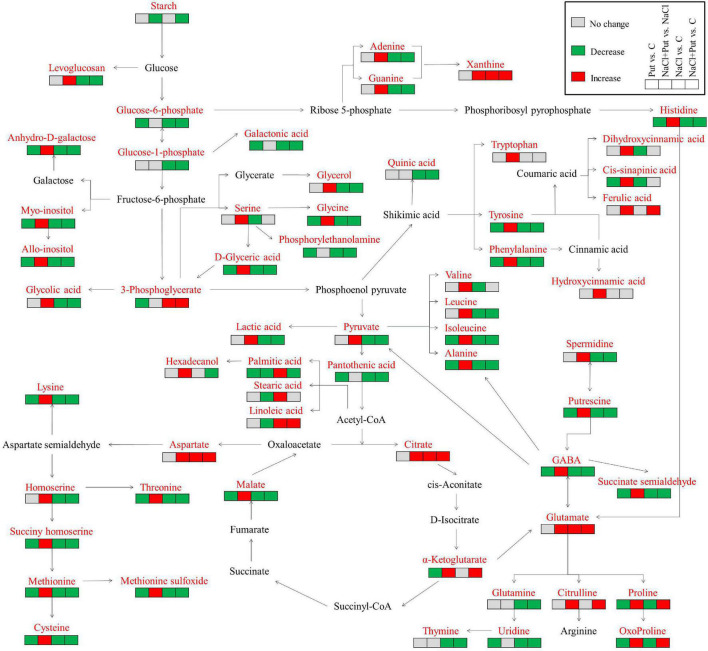
An assessment of 60 metabolites in the map of metabolic pathways. Red, green, and gray mean the significant increase, decrease, and unchanged, respectively.

Metabolic pathways in association with sugars and AAs metabolism, TCA cycle, and g-aminobutyric acid (GABA) shunt were shown in Figure 8. A total of 60 out of 102 identified metabolites (23 AAs, 4 sugars, 13 organic acids, 5 lipids and fatty acids, 3 alcohols, and 12 other metabolites) were assigned to metabolic pathways. The application of Put had a pronounced effect on salt-stressed seedlings (NaCl+Put vs. C) relative to non-stressed seedlings (Put vs. C). Put-induced metabolites accumulation was mainly involved in amino acid and organic acid metabolism under salt stress. The Put-pretreated seeds maintained significantly higher contents of intermediate metabolites involved in the TCA cycle and GABA shunt than the water-primed seeds (NaCl+Put vs. NaCl) during germination under salt stress (Figure 8).

## Discussion

Seed germination experiences a series of complex physical and physiological processes including water absorption, activation and formation of enzyme systems, and mobilization of reserve substances such as starches, proteins, fatty acids, and minerals for seedling establishment and growth. Starches are the main energy reserves closely related to seed germination and stress resistance (Yang et al., 2016). During seed germination, starches in the endosperm are rapidly hydrolyzed into monosaccharides and other small molecular substances by various amylases. As one of the most important hydrolysates of starch, the glucose is metabolized into glycolysis and pentose phosphate pathways to provide energy for seed germination (Smeekens et al., 2010). In addition, soluble sugars also act as important osmolytes and osmoprotectants performing crucial roles in the maintenance of water balance and the integrity of lipids membrane structure during germination under salt stress (Boriboonkaset et al., 2013). A previous study has shown that salt stress significantly decreased germination and embryo growth of white clover seeds associated with the significant inhibition of starch catabolism or sugars metabolism (Cheng et al., 2018). It has been found that Put could significantly promote the metabolism of energy storage substances such as proteins and starches contributing to enhanced seed germination and seedling growth of rice under salt stress (Prakash et al., 1988). Here, our study showed that salt stress significantly decreased total amylase and β-amylase activities in white clover seedlings without the Put pretreatment. However, Put-treated white clover seedlings exhibited significant increases in total amylase, α-amylase, and β-amylase activities together with a significant reduction in starch content under salt stress (Figure 3). The metabolomic analysis also demonstrated that Put effectively alleviated salt stress-induced declines in levoglucosan, fucose, and anhydro-D-galactose contents during seed germination (Figure 7B). These findings indicated that Put could activate amylases to accelerate starch hydrolysis contributing to improved soluble sugars for seed germination and seedling establishment under salt stress (Figure 3). Our current findings were consistent with the study of Çavuşoğlu et al. (2007) who reported that Put pretreatment significantly improved barley seed germination and seedlings establishment under salt stress.

During seed germination, smaller molecules “monosaccharides” from starch hydolysis undergo glycolysis to produce pyruvate perform vital functions in the TCA cycle and mutual transformation of sugars, proteins, and lipids (Min et al., 2016; Zhang et al., 2016). Salt stress significantly reduced the production of pyruvate and intermediates of the TCA cycle such as citric acid, α-ketoglutaric acid, succinic acid, and malic acid, thereby inhibiting seed germination and subsequent seedling establishment (Zhang et al., 2016; Cheng et al., 2018). In our current study, the decrease in total organic acid content caused by salt stress was observed in white clover seedlings, whereas the total organic acid content was maintained at a high level in seeds soaked with Put under salt stress (Figure 5C). The Put pretreatment also significantly alleviated salt-induced reductions in pyruvate, citric acid, α-ketoglutaric acid, and malic acid during white clover seed germination, which indicated better maintenance of TCA cycle in seedlings in response to salt stress (Figure 7A). An earlier study by Min et al. (2016) found that exogenous Put significantly increased accumulations of organic acids and pyruvate, hence promoting the release of more energy currencies (ATP and ADP) in leaves associated with improved salt tolerance of cucumber (*Cucumis sativus*) seedlings.

In addition to metabolic roles for energy supply, organic acids play critical roles in maintaining ionization equilibrium, antioxidant defense, and other functions in cell saps due to their property of weak acid (Lin et al., 2014). Glycolic acid is an important intermediate metabolite of the photorespiration pathway in higher plants (Hong et al., 2017). The inhibition of glycolic acid metabolism reduces photorespiration rate and photosynthetic rate, resulting in reduced glycine and serine content, growth retardation, and even “respiratory condition death” (Bai et al., 2014; Lu et al., 2014). Significantly higher glycolic acid content was only observed in the Put-pretreated white clover seedlings after 7 days of germination under salt stress, indicating that the Put-induced salt tolerance could be related to the glycolic acid pathway (Figure 7A). Previous studies have shown that phenolic acids such as caffeic acid (dihydroxycinnamic acid), sinapinic acid, and ferulic acid with strong antioxidant capacity positively regulated plant nutrient absorption, enzyme activity, and photosynthesis (Faulds and Williamson, 1999; Robbins, 2003). It has been reported that dihydroxycinnamic acid with appropriate concentration could significantly improve the salt tolerance of soybeans and the drought tolerance of cucumber seedlings (Klein et al., 2013; Wan et al., 2014). The productions of sinapinic acid and choline in the endosperm were conducive to seed germination (Strack, 1981). The exogenous application of sinapinic acid promoted seed germination of *Arabidopsis* and partially restored the germination inhibition induced by ABA (Bi et al., 2017). Ferulic acid is one of the most effective components of medicinal plants such as Ferula (*Ferula asafoetida*) and Angelica (*Angelica sinensis*) associated with systematic stress tolerance (Lu et al., 2005; Aybek et al., 2018). In our current study, Put-pretreated white clover seedlings maintained significantly higher dihydroxycinnamic acid, sinapinic acid, and ferulic acid levels than water-pretreated seedlings, indicating that the Put-enhanced salt tolerance could be related to the phenolic acid pathway during seed germination.

Based on the metabolic profiling analysis, salt stress significantly decreased all detected AAs in white clover seedlings, except for the aspartic acid, glutamic acid, citrulline, and aminoisobutyric acid (Figure 6). These various AAs can be used as key regulators of osmotic potential, antioxidant defense, energy metabolism, or signal molecules in cells contributing to stress tolerance in plant species (Hu et al., 2015; Shi et al., 2015; Li et al., 2016; Xu et al., 2018). It has been proved that enhanced salt tolerance induced by Put was related to the accumulation of many AAs such as threonic acid, serine, aspartic acid, histidine, lysine, alanine, isoleucine, norvaline, and proline in cucumber (Yuan et al., 2016). In this study, white clover seeds soaked with Put exhibited significantly higher total AAs content and individual amino acid content including proline, glutamate, cysteine, aspartic acid, tyrosine, methionine, serine, threonine, alanine, GABA, etc. than the seeds without the Put pretreatment, which indicated that the mediation of AAs might play an important role in the Put-induced salt tolerance during seed germination. Proline is one of the most abundant AAs in plants (Figure 6). Rapid accumulation of the proline improved osmotic adjustment (OA) and antioxidants capacity when plants responded to abiotic stress (Ashraf and Foolad, 2007; Signorelli et al., 2013). Glutamate is involved in the synthesis of other AAs such as GABA, glutamine, citrulline, proline, and 5-oxopropyl in plants and also participates in nitrogen metabolism under stress conditions (Bouché and Fromm, 2004). In addition, it is well known that cysteine has a strong antioxidant effect in alleviating stress-induced oxidative damage in plants (Youssefian et al., 2001). Put-regulated salt tolerance was related to the transformation of Put into the synthesis pathway of GABA, glutamate, and proline in different plant species (Gill and Tuteja, 2010; Min et al., 2016). In this study, Put enhanced salt tolerance during seed germination by promoting proline, glutamate, cysteine, and GABA accumulation and metabolism (Figure 6).

Methionine is a sulfur-containing amino acid that not only participates in the anabolism of ethylene and PAs (Spd and Spm) but also exhibits a strong antioxidant effect in plants (Jia et al., 2009). Plastoquinone and ubiquinone are important intermediate transmitters in the plant electron transport chain (Shukla and Dubey, 2018). Tyrosine is involved in the production of metabolites such as tocopherol, plastoquinone, ubiquinone β-galactoside, salidroside, and benzylisoquinoline alkaloid which are essential for plant survival (Xu et al., 2020). Aspartic acid is a precursor of plant essential AAs including lysine, threonine, methionine, and isoleucine (Azevedo and Lea, 2001). In addition, aspartic acid also participates in the transportation and storage of nitrogen in plants (Azevedo et al., 2006). Serine is involved in the photorespiration pathway, 3-phosphoglycerate pathway in plastids, and C_1_ tetrahydrofolate synthase/serine hydroxymethyltransferase pathways (Zhu et al., 2004). It has been reported that alanine alleviated salt-induced inhibition of various enzymes involved in nitrogen fixation, photosynthesis, and respiration (Thomas and Shanmugasundaram, 1991). Therefore, the Put-induced accumulation of diverse AAs including methionine, alanine, serine, tyrosine, and aspartic acid could be associated with important roles in metabolic homeostasis, OA, and antioxidant during seed germination of white clover under salt stress (Figure 6).

According to our current findings, salt stress or salt stress in combination with the Put priming significantly induced alterations in glycerol, fatty acids, alcohols, and other metabolites during seed germination. Fatty acids and glycerol are metabolized into the TCA cycle to release energy for seed germination (Manuel et al., 2013). It has been found that enhanced hydrolysis and conversion of glycerol and fatty acids promoted aged soybean (*Glycine max*) seed germination and seedling establishment (Zhou et al., 2018). Our findings showed that the glycerol content significantly decreased, but fatty acids (stearic acid, linoleic acid, and palmitic acid) content significantly increased in geminated white clover seeds subjected to salt stress (Figure 7C). Interestingly, the Put-pretreated white clover seeds maintained significantly lower stearic acid, linoleic acid, and palmitic acid contents than the seeds without the Put pretreatment after 7 days of germination under salt stress, which could indicate that these fatty acids might have been used for seedling establishment. The previous study has proved that increased metabolism and conversion of fatty acids to sugars regulated by diethyl aminoethyl hexanoate were beneficial to the germination of aged soybean seeds (Zhou et al., 2018). In addition, the Put pretreatment significantly alleviated salt-induced declines in the myo-inositol, allo-inositol, hexadecanol, threitol, hydroxylamine, formononetin, adenine, and guanine when white clover seeds germinated under salt stress (Figures 7B,C). Inositol acts as precursor and substrate for the biosynthesis of many important metabolites such as inositol hexaphosphate, phosphatidylinositol, galactitol, ascorbic acid, indoleacetic acid conjugates, ononitol, and pinitol which have diverse functions in plant growth and adaptation to stress (Stevenson et al., 2000; Donahue et al., 2010; Saxena et al., 2013). The overexpression of a *CaIMP* gene in *Arabidopsis* associated with inositol accumulation significantly improved seed germination and seedling growth under lithium stress (Saxena et al., 2013). Adenine is converted into adenine monophosphate (AMP) in plant tissues to increase the concentration of ATP and plant growth. It has been found that the application of guanine and adenine significantly improved the growth and salt tolerance of wheat (*Triticum aestivum*) (Hussein et al., 2015). Significantly higher adenine and guanine contents were observed in the Put-pretreated white clover seedlings after 7 days of germination under salt stress, indicating that the Put-enhanced salt tolerance was related to the purine metabolic pathway (Figure 7C). Formononetin is an important member of flavonoids exhibiting an obvious antioxidant effect in plants and its accumulation helps to alleviate oxidative damage caused by abiotic stresses (Saviranta et al., 2010; Fini et al., 2011; Li et al., 2017). Our results showed that seeds soaking with Put exhibited significantly higher formononetin content than the seeds soaked with water, which could be associated with the maintenance of ROS homeostasis during white clover seed germination under salt stress (Figure 7C).

Under salt stress, excessive accumulation of Na^+^ causes ionic toxicity resulting in an ionic and metabolic imbalance in plant cells (Gong et al., 2020). For example, a large amount of Na^+^ in cells not only blocks K^+^ absorption but also accelerates K^+^ loss and the production of ROS which damages the structure and function of the cell membrane during germination of white clover seeds under salt stress (Cheng et al., 2018). Salt stress inhibited melatonin accumulation and also enhanced ABA signal transduction to promote the accumulation of ROS, resulting in oxidative damage and reduced seed germination (Luo et al., 2020; Chen et al., 2021). Our current results showed that more than forty times higher Na^+^ content was detected in the salt-stressed white clover seedlings as compared with that in non-stressed seedlings. However, the Put application not only significantly inhibited Na^+^ accumulation but also significantly alleviated salt-induced K^+^ loss resulting in a lower Na^+^/K^+^ ratio in white clover seedlings (Figures 4A–C). These findings indicated that the Put-regulated white clover seed germination and subsequent seedlings establishment are associated with changes in Na^+^ and K^+^ transport and distribution under salt stress (Figure 4). It has been found that *SOS1* is very important for Na^+^ homeostasis in plants. For example, the study of Shi et al. (2000) proved that the *SOS1* mutant *Arabidopsis* seedlings were highly sensitive to Na^+^, and overexpression of *AtSOS1* in the *SOS1* mutant significantly reduced the sensitivity to Na^+^. The study of Yadav et al. (2012) found that *SbSOS1* could transport Na^+^ from the phloem to the xylem resulting in reduced Na^+^ toxicity in living cells. Exogenous Spm significantly up-regulated the *AsSOS1* expression associated with maintenance of low Na^+^ content and higher K^+^/Na^+^ ratio in leaves and roots when creeping bentgrass suffered from salt stress (Geng et al., 2021). Significant up-regulation of *SOS1* and *H^+^-ATP* expression levels was observed only in the Put-pretreated white clover seedlings after 7 days of germination under salt stress indicating that the Put-enhanced salt tolerance was related to the SOS pathway (Figure 4).

In addition, salt stress significantly reduced the expression levels of *NHX6*, *SKOR*, *HKT1*, *HKT8*, and *HAL2* gene in white clover seedlings, however, seeds primed with Put could significantly alleviate these phenomena (Figure 4D). NHX6 regulates the balance of Na^+^ and K^+^ in reticular structures of golgi bodies (Bassil et al., 2011). A previous study found that the K^+^ content in *nhx5*/*nhx6* double mutant *Arabidopsis* plants was significantly lower than that in wild-type plants, which could be eliminated by restoring the expression of *AtNHX5* or *AtNHX6* in double mutant plants (Wang et al., 2015). SKOR is a key protein for the long-distance distribution of K^+^ from root to upper parts of the plant and contributes up to 50% of K^+^ transport in xylem sap (Huang et al., 2018). Under salt stress, ZmHKT1 or OsHKT8 mediate the Na^+^ rejection in xylem sap or phloem, respectively, and both of them prevent the transfer of Na^+^ to young leaves. Impaired performance of *hkt1* or *hkt8* increased Na^+^ concentration in aboveground tissues leading to reduced salt tolerance in plants (Kobayashi et al., 2017; Zhang et al., 2018). Our results showed that transcript levels of *HKT1* or *HKT8* in seeds primed with Put was 6.20 or 4.94 times higher than that in seeds soaked in water under salt stress (Figure 4D). These findings demonstrated that the maintenance of higher expression levels of *NHX6*, *SKOR*, *HKT1*, and *HKT8* induced by the Put could play critical roles in Na^+^ rejection and K^+^ retention during white clover seed germination (Figure 4D). In addition, HAL2 hydrolyzed intermediate product 3′-phosphoadenosine-5′-phosphosulfate (PAPS) in the process of sulfur assimilation in rice and *Arabidopsis* cells under salt stress, which reduced PAPS toxicity in cells (Gil-Mascarell et al., 2010). Put-pretreated white clover seedlings exhibited significantly higher *HAL2* expression under salt stress, which could enhance detoxification ability to PAPS (Figure 4D).

## Conclusion

**FIGURE 9:**
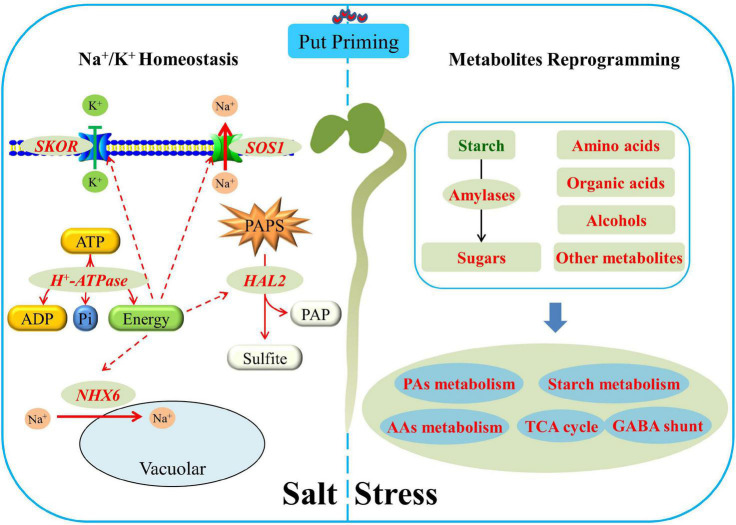
Integrative pathways regulated by the Put priming during white clover seeds germination under salt stress. Red or green letters mean a significant increase or decrease, respectively.

Seed soaking with Put significantly alleviated salt stress-induced inhibition of white clover seed germination and subsequent seedlings establishment associated with reduced oxidative damage and accelerated amylolysis. The metabolomic analysis found that the Put-pretreated seeds maintained significantly higher AAs, organic acids, sugars, and other metabolites contents than non-pretreated seeds after 7 days of germination under salt stress. Put promoted sugar and AAs metabolism, TCA cycle, and GABA shunt associated with better cell membrane stability, energy supply, and metabolic homeostasis when seeds germinated under salt stress. Put-induced increases in other metabolites accumulation such as myo-inositol, guanine, adenine, and formononetin could be involved in enhanced antioxidant defense and osmotic adjustment contributing to salt tolerance. Under salt stress, Put significantly up-regulated the *AsSOS1*, *NHX6*, *SKOR*, *HKT1*, and *HKT8* expression levels which played critical roles in Na^+^ rejection and K^+^ retention resulting in a higher K^+^/Na^+^ ratio during seed germination. In addition, Put-induced up-regulation of *HAL2* transcription level could reduce the toxicity of PAPS in cells (Figure 9). Current findings provide an integrative understanding of Put-induced salt tolerance associated with amylolysis, metabolic regulation, and ionic homeostasis during seed germination.

## Data Availability Statement

The datasets presented in this study can be found in online repositories. The names of the repository/repositories and accession number(s) can be found in the article/supplementary material.

## Author Contributions

ZL and YP designed the experiments and improved the manuscript. BC performed the experiments and wrote the manuscript. MH, JZ, WL, and GF provided materials and editorial advice and also improved the manuscript. All authors contributed to the article and approved the submitted version.

## Conflict of Interest

The authors declare that the research was conducted in the absence of any commercial or financial relationships that could be construed as a potential conflict of interest.

## Publisher’s Note

All claims expressed in this article are solely those of the authors and do not necessarily represent those of their affiliated organizations, or those of the publisher, the editors and the reviewers. Any product that may be evaluated in this article, or claim that may be made by its manufacturer, is not guaranteed or endorsed by the publisher.
